# Multi Scale Ethics—Why We Need  to Consider the Ethics of AI in Healthcare at Different Scales

**DOI:** 10.1007/s11948-022-00396-z

**Published:** 2022-11-28

**Authors:** Melanie Smallman

**Affiliations:** Alan Turing Institute & Department of Science and Technology Studies, University College London, Gower Street, London, WC1E 6BT UK

**Keywords:** Ethics, Scale, Coproduction, STS, Data ethics, Multiscale ethics framework, Mulitscale ethics, Group effects

## Abstract

Many researchers have documented how AI and data driven technologies have the potential to have profound effects on our lives—in ways that make these technologies stand out from those that went before. Around the world, we are seeing a significant growth in interest and investment in AI in healthcare. This has been coupled with rising concerns about the ethical implications of these technologies and an array of ethical guidelines for the use of AI and data in healthcare has arisen. Nevertheless, the question of if and how AI and data technologies can be ethical remains open to debate. This paper aims to contribute to this debate by considering the wide range of implications that have been attributed to these technologies and asking whether current ethical guidelines take these factors into account. In particular, the paper argues that while current ethics guidelines for AI in healthcare effectively account for the four key issues identified in the ethics literature (transparency; fairness; responsibility and privacy), they have largely neglected wider issues relating to the way in which these technologies shape institutional and social arrangements. This, I argue, has given current ethics guidelines a strong focus on evaluating the impact of these technologies on the individual, while not accounting for the powerful social shaping effects of these technologies. To address this, the paper proposes a Multiscale Ethics Framework, which aims to help technology developers and ethical evaluations to consider the wider implications of these technologies.

## Introduction

In May 2018, a group of my neighbours dressed up in bandages and fake wounds and lay on the ground in our local town square. They were staging a public ‘die-in’, as a dramatic protest against the ‘GP at Hand’ AI app, described as the first instance of AI being used within the UK’s National Health Service (NHS) (Murgia, 2017). A few weeks earlier, a similar protest had taken place at the GP at Hand facility in East London. Protestors carried placards declaring “GP at Hand should be Banned” and “GP at Hand. Not in my land!”.

The ‘GP at Hand’ app was being piloted in my local ‘Clinical Commissioning Group’ (CCG)—the area-based organisation responsible for planning local healthcare services. Initially launched in April 2018 with the slogan “See an NHS GP in minutes, for free 24–7” (Babylon Health, 2018), the £21 m bill for the App’s service was now being cited in CCG board papers as a key driver of the service cuts that were being proposed, as the CCG tried to balance the books—cuts that would see GP surgeries in my neighbourhood being forced to reduce the number of morning, evening, and weekend appointments they offered (Iacobucci, 2019). While government ministers and technologists declared the promise of AI to reduce costs and increase productivity and accuracy in the NHS, local service users could see a different story. As one protestor put it “They are taking NHS money away from GP surgeries who need it to take care of the old and sick. This is bad news for everyone—except GP at Hand.” (Bostock, 2018). This early implementation of AI in the UK’s NHS was shaping the health service in very particular ways—and my neighbours were not happy.

While this protest could be seen as a protest against a particular policy rather than against AI, many researchers have been documenting how AI is having particularly profound effects on people’s lives, making it uniquely difficult to separate the technologies from their social effects, to such an extent that it is only worthwhile to understand them as socio-technical systems. For instance, Brynjolfsson and McAfee (2014) have described the scale of transformation brought about by AI and digital technologies as being as significant to human progress as was the invention of the steam engine. Specifically, they argue that that vast and unprecedented boost to mental power offered by these advanced technologies will affect human progress as much as the boost to physical power offered by the steam age; Eubanks,(2017) and Benjamin, (2019) have explained how the seeming objectivity of these technologies, and the way in which they become embedded and black-boxed inside service delivery, is increasing and embedding historic inequalities in troubling ways; Susskind, (2018) points out that digital technologies create new moral dilemmas—such as what is acceptable behaviour in virtual spaces; and Smallman (2019) has pointed out that the vast cost of these technologies limits the possible shape of public services in the future. Jasanoff, (2004) goes as far as to argue that the ways we know and represent the world (in this case through technology and data) are inseparable from the ways in which people seek to control and organise the world, pointing us to look at the wider social orders that are enabled or disabled by these technologies if we want to fully understand their effects.

In keeping with this transformative power of AI and data technologies, the past few years has seen the growth in interest and investment in AI in healthcare, coupled with rising concerns about the ethical implications of these technologies (Hateley, 2017; Health, 2020; Schwalbe & Wahl, 2020; Vollmer et al., 2020) and matched by an array of ethical guidelines for the use of AI and data in healthcare (Jobin et al., 2019). For instance, UNESCO’s International Bioethics Committee has produced a report Big Data and Health (UNESCO, 2017) and the UK Department of Health & Social Care (the government department overseeing the NHS) has issued a ‘Code of conduct for data-driven health and care technology’ (Department of Health & Social Care, 2018). Yet even with these ethical guidelines in place, my neighbours were still driven to the streets to protest against these technologies. This raises the question of whether and how their concerns were accounted for in these ethical guidelines.

In this paper, I argue that we need to rethink how we govern and evaluate advanced technologies like AI, in order to take account of the wider social effects that they bring with them—and that appear to be driving public responses to these technologies. Specifically, I argue that firstly, we need to understand that advanced technologies are not simply tools or solutions, but bring particular ways of understanding and organising the world, and so evaluation approaches that seek to separate the technologies from their social settings and effects will be insufficient. Secondly, with this social ordering effect in mind, we need to develop a way of understanding and evaluating technologies that look beyond their effect on individuals, to account for their wider effects on institutions and societies. To do this, I propose a Multi-Scale Ethics (MSE) approach that enables technology developers and policymakers to anticipate and take account of the effects of AI^1^ at different scales—from its impact on the individual, to communities, the planet and future generations. Rather than analysing the technology as an entity that is separate (or separable) from the way it is used (as I have previously argued technology developers often do (Smallman, 2017), and focusing on an individual rights based approach to ethics (as I will show current ethics guidelines do), the framework accepts AI and digital technologies as socio-technical systems, which produce both technologies and particular social arrangements. Taking this collective entanglement as the unit of analysis, I will argue, allows for a much richer understanding of the concerns raised by these technologies—and therefore a much greater change of anticipating and addressing them.

Importantly, while I make an argument based on evidence gathered studying AI in healthcare settings, and although the points here are particularly pertinent (and pressing) for the UK’s nationalised healthcare system, the issues raised and the multiscale ethics framework proposed are likely to be relevant to advanced technologies more widely. I reflect on that more fully in the discussion.

## The Impact of AI in Healthcare

Alongside the growing interest in the potential of AI to transform healthcare, the past five or so years has seen the development of various guidelines for the ethical use of AI, as well as a handful focused on AI in healthcare specifically. The wider set of guidelines for the ethical use of AI in general have been subject to a number of reviews in the past two years. Although the guidelines reviewed did not focus on healthcare specifically, they appear to form a high level framework for other, future and more field-specific guidelines to draw upon and sit within. These reviews have highlighted a strong convergence around five key ethical principles: transparency; justice and fairness; non-maleficence; responsibility & accountability; and privacy (Fjeld, et al., 2020; Hagendorff, 2020; Jobin et al., 2019). Hagendorff (2020) found that 80% of the guidelines reviewed contained reference to three of these principles in particular—accountability, privacy and fairness. I will reflect upon the reasons for the convergence on these particular topics later, but four things are clear from these analyses: Firstly, current AI ethics guidelines are strongly driven by a rights based approach to ethics; secondly, and perhaps as a consequence of this rights based approach, the focus is on the effect of technologies on the individual; thirdly, the guidelines assume that ‘ethically sound’ AI systems are possible, which brings the implication that ethical issues are epiphenomena which can be addressed if the ‘right’ ethics approach is in place; and fourth, again perhaps flowing from this rights based approach, the power of AI technology to shape social arrangements in particular way, are neglected. Hagendorff (2020) sums up these omissions: “Almost no guideline talks about AI in contexts of care, nurture, help, welfare, social responsibility or ecological networks. In AI ethics, technical artefacts are primarily seen as isolated entities that can be optimized by experts so as to find technical solutions for technical problems. What is often lacking is a consideration of the wider contexts and the comprehensive relationship networks in which technical systems are embedded”.

Elsewhere an analogy between AI and cars has been drawn (Smallman, 2019): Thinking about cars as modes of transport is akin to thinking about AI as simply a set of tools, with their effects being the result of decisions about their use. But we just have to look out of our windows to see how cars have shaped the very fabric our lives. Most decisions we make—from where we live, who we spend time with, to where we work and what we eat—have been shaped by the car. Cars open up some possibilities and make others less possible, affecting possible options even for those of us who don’t drive cars. And decisions made at one level can have significant and sometimes unexpected effects on other levels (for instance, whole neighbourhoods suffering poor air quality because of individuals choosing to drive). It would not be an exaggeration to describe cars as one of the biggest shapers of our world in the twentieth century. Evaluating them as modes of transport—or assuming their effects are going to be felt entirely by those who interact with the technologies—is wholly insufficient if we are genuinely concerned about their moral and ethical implications. Echoing this, Morley et al., (2020) have highlighted how, even without looking at the sociological effects of advanced technologies, ethical effects might change at various “levels of abstraction”. Ethical concerns move from being matters of moral responsibility for individuals to being ones of liability for institutions or sectors. Beyond this, in their work on multi-lifespan information systems, Friedman et al. (2017), Friedman and Nathan (2010) have argued that many of the challenges being addressed by advanced technologies defy rapid solutions and require long periods of time to unfold. As such, longer term and diverse perspectives need to be taken into account in the development of such technologies.

Thinking about this more expansive role of AI—as a driver of structural change rather than just a tool—is likely to be a helpful way to consider the effects of AI in healthcare, as evidence has been growing that rather than simply transforming the efficiency of treatment and the patient experience, advanced technologies like AI and robotics present powerful forces of much wider change. For example, studies of robotics have found that the vast cost of the technologies means that healthcare provision needs to become more centralised (Aggarwal et al., 2017; Zietman, 2018). This is often at the expense of more local, traditional care, resulting in poorer access to health care for lower income households who tend to have less access to transport, potentially deepening further existing health inequalities (Aggarwal 2017). Added to that, He et al. (2019) described how a virtuous circle is set up by investments in labour-saving technology, as the falling labour costs free up more money for future investments, concentrating wealth in already wealth NHS trusts. The benefits from such advanced technologies are not shared equally across populations and communities, and the way that these benefits pattern appear to be significant ethical issues that are currently overlooked in most ethical evaluations of these technologies. Importantly, for nationalised healthcare systems working in democratic states—such as the UK’s National Health Service (NHS)—if technologies are driving the shape of service provision in particular directions (or at least offering affordances that tend towards one particular shape of service and close off other possibilities), further ethical questions arise around the power of decisionmakers to intervene and be accountable for the shape of service provision, and for citizens to scrutinse and affect these decisions. This is all the more important when these affordances are coupled with data driven health resource allocation, which, as (O’Doherty et al., 2016) point out, are not simply technical steps but depend on values to decide how data is used (eg judging what kinds of behaviour qualify has high risk), itself a step that should, but is difficult to, be subject to democratic scrutiny.

The economics of advanced technologies also raises important ethical questions relating to the affordability of healthcare services in the future and ultimately the role of these technologies in creating a better future for humankind. Firstly, there is growing evidence that hi-tech innovation has played a role in driving inequality in developed nations (Brynjolffson & McAfee, 2014, p. 168). Hi-tech innovations have a tendency to concentrate wealth in the hands of those with the most assets and capital (Aghion et al., 2015); they also have a tendency to drive up demand and wages amongst high-skill, hi-tech (and typically male) workers, while undermining job security for low-skilled workers (Acemoglu & Autor, 2010; Autor et al., 1998; Birch et al., 2020; Cozzens, 2012; Cozzens et al., 2002; Rogers, 2003). Connecting these trends to previous work looking at the links between social inequality and poor physical and mental health (Kondo et al., 2009), it is possible to see that while investing in AI technologies might benefit particular individuals receiving hi-tech treatment, improving the health of the population as a whole could prove more, not less difficult by significant investments in hi-tech healthcare.

Secondly, adopting advanced technologies usually involves the transfer of assets (money and or data) from publicly funded healthcare services to private corporations. In the simplest terms, for the NHS, this means a technology-driven privatisation of services. This should be more than an ideological concern: Where the money goes matters for the sustainability of public services reliant on tax revenue and for services aiming to improve health outcomes. Alongside the work above showing that hi-tech innovations tend to concentrate wealth into the hands of the already wealth, Brynjolfsson and McAfee, (2014) have described how digital technologies have brought in a particular business model that (a) enables services to be delivered with significantly fewer staff, and (b) enables products and services to be produced and delivered from anywhere in the world, creating the phenomenon of “stateless profit”. Under this model, investment in advanced technologies could mean that public NHS funds get diverted from paying staff (who provide a return back to the public purse through income and sales taxes) and instead to international corporations, who are better  placed to avoid making a return to the public purse—ultimately undermining the state’s ability to fund healthcare provision for future generations.

As well as reconstituting healthcare institutions and economic structures, widespread adoption of advanced technologies in healthcare also has the potential to reconstitute relationships between healthcare providers, patients, citizens and states. Lupton (2014) has described how ideas of the ‘ideal patient’ have shifted in the digital era, such that the ideal patient now accesses relevant information and monitors their own health, both in the interests of preserving and promoting their own good health and to relieve the financial burden on the healthcare system in the current era of cost savings. With the advent of data driven and AI healthcare technologies, he argues that the idea of a ‘digitally engaged citizen’ is now growing to include making use of digital technologies in order to provide information to other patients and healthcare providers. In the digital era, a good patient is also a willing data subject, prepared to digitise and survey themselves, and to share that digital information. O’Doherty et al (2016) point out that information collected in health research clearly has implications for family members and ethnic groups to which individuals belong—now and in the future. Furthermore, far from minoritized groups and low-income households being excluded from datasets and online services, as suggested in a number of the reports reviewed below, research to date indicates that these groups are likely to be subject to increased datafication and surveillance, to such an extent that Eubanks (2017) has coined the phrase ‘digital poorhouse’, describing how the lowest income households are forced to give up their privacy in return for state support. Benjamin (2019) highlights how particular racial groups are heavily over-represented in certain data sets and have more frequent engagement with a number of data-driven algorithms, not necessarily for positive reasons or for their benefit. Birch et al., (2020) have argued that issues such as this, surrounding “privacy, consent, and behavioural influence implied in the centralized control of personal data by a few, monopolistic “Big Tech” companies”, are of growing concern for a range of political and civil society voices right now.

Beyond healthcare specifically, the wider literature about the social impacts of AI highlights significant concerns about its potential to disrupt democratic and social structures in a potentially troubling way. Friedman et al. (2017), Friedman and Nathan (2010) have described the importance of understanding the development and effects of technologies over multiple generations or lifetimes. In an essay in the journal Science (Zwitter et al., 2017), a group of nine scientists went as far as to pose the question "Will Democracy Survive Big Data and Artificial Intelligence?" stating “one thing is clear: The way in which we organize the economy and society will change fundamentally…If we take the wrong decisions it could threaten our greatest historical achievements” (Zwitter et al., 2017). Starting with examples of the current data-driven or surveillance capitalism—where private companies use your browsing history to recommend (and thereby arguably automate) shopping choices—they fast forwarded, placing this technology into the hands of governments wanting to ensure citizens do the ‘right’ thing (for instance steering citizens towards healthier lifestyles). This might appear to be a positive step forwards, but the authors argue that coupling the uncertainty of these advanced technologies with a lack of transparency and insufficient democratic control, gives these technologies the potential to destroy social cohesion and to increase conflict, as different groups exist within information bubbles and no longer understand each other. Even more worryingly, they suggest that if data driven technologies made decisions without accounting for the cultural and social cues that are currently important, they have the potential to cause serious social damage, including discrimination, extremism and conflict. Many of the most serious implications of healthcare AI are likely to be emergent, with the harshest effects felt by communities with the least powerful voices (Smallman, 2019).

There appears to be a strong need to develop a way to incorporate these powerful social-shaping effects of advanced technologies into ethical guidance and evaluations then. But in the first instance it is important to know whether or not current guidelines for the ethical use of AI in healthcare maintain the focus on the five issues identified in reviews of high-level guidelines for ethical AI in general, or, given the particular consequences and patient focus of healthcare, do healthcare specific guidelines begin to take account of these wider ‘sociological’ concerns?

## Ethics of AI in Current Guidelines for AI and Data Technology in Healthcare

### Methods

To understand whether and how current guidelines for the ethical use of AI in healthcare address these sociological effects, a comprehensive database of guidelines for ethical AI in Healthcare was developed through:

- A systematic online search
- Recommendations from academic and practitioner experts
- Reviews of other relevant literature (detailed above)

This process identified seven sets of guidelines, each of which were downloaded and read, in order to verify that they were sets of guidelines and did relate to the ethical use of AI and data technology in healthcare specifically. All seven were included in the review (and listed in appendix 1). That there weren’t more guidelines of this sort was surprising and perhaps points towards the importance of the ‘general’ guidelines for ethical AI, in providing high-level guidance to date.

Once the corpus had been produced, the guidelines were analysed using qualitative content analysis which involves searching-out underlying themes in the materials being analysed (Bryman, 2012). The reports were coded using a framework based upon (a) the principles of ethical AI found in the existing literature (described above) and (b) the possible levels on which the expected sociological effects are likely to work, based on the Science and Technology Studies (STS) literature (also described above):

### Coding Framework

1. Transparency;
2. Justice and fairness;
3. Non-maleficence;
4. Responsibility & accountability;
5. Privacy
6. The individual
7. Groups and communities
8. The Institution/NHS
9. The Nation
10. Globally
11. Over time

### Analysis of Existing Guidelines for AI and Data Technology in Healthcare

So do the current guidelines for the ethical use of AI in healthcare maintain the focus on the five issues identified in reviews of guidelines for ethical AI in general, or do these healthcare specific guidelines begin to take account of the wider ‘sociological’ concerns?

Looking at these frameworks for AI in healthcare, it becomes apparent that while the precise language varies, as with the guidelines for general AI they too cluster around four of the five concerns identified in the previous reviews of AI ethical guidelines: transparency; fairness; responsibility; and privacy. Interestingly, non-maleficence was not mentioned in any of the reports, which is perhaps because the aim of doing no harm was seen as a given in a healthcare context.

Besides these four ‘core’ concerns, issues relating to bias, technical robustness and public trust appear to be significant and highlighted in most of the guidelines. Typically, they are viewed together and seen to be a technical issue about the veracity and reliability of the data underpinning the apps, such that solutions to questions of bias and trust are seen as technical matters that can be solved with better data and the right apps. For instance, the Wellcome Trust (2018) report considers what makes algorithms trustworthy, arguing that trustworthiness is based upon three factors: the effectiveness of the technology; where the technology comes from and who developed it; and what kind of data it was trained on (page 41); similarly the EIT (2019) report recommends that asking app developers to use data sheets to document data sources would be a key way of ensuring transparency and auditability; the Department of Health and Social Care (2018) gives similar emphasis, recommending regular assessments of data quality.

In terms of the underlying approaches taken by these guidelines, throughout, the reports all tended to give normative statements about how ethical AI should look. For instance, the Nuffield Council for Bioethics (2015) gives a series of recommendations for the ethical use of data, stating that “The use of data in biomedical research and health care should be in accordance with a publicly statable set of morally reasonable expectations and subject to appropriate governance.” Similarly, the Department of Health & Social Care Code of Conduct for data-driven health and care technology gives 10 ‘Principles’.

It also clear that as with mainstream AI ethics, a rights-based approach has been adopted across the range of guidelines. For example, the UNESCO report cites the UN Universal Declaration on Human Rights (1948) and the European Convention on Human Rights in framing its approach to the issue of Big Data ethics (page 4). Similarly, the Nuffield Council on Bioethics report cites human rights as a framing for discussion on issue of privacy. Less specifically, the EIT/Microsoft report refers to the potential of AI to “negatively affect fundamental rights”, while the Department of Health & Social Care Code of Conduct refers to the principle of “respecting human rights”. Some discussions of utilitarian approaches are evident when group effects, such as the public good, are discussed. For instance, the Nuffield Council on Bioethics report discusses the philosophies of John Stewart Mills and the challenge of accounting for what is the greatest good. This is however a side-bar within a report that is more directly underpinned by rights-based ideas.

There are very important historic reasons for this focus on rights that go back to the foundations of bioethics and the need to protect citizens from oppressive and coercive states in the mid to late twentieth century Europe. While this protection is still vital, one of the consequences of this rights- based approach is that, throughout the guidelines reviewed, the scale of focus is almost exclusively on the individual. Groups and communities are mentioned in a number of places, but these group-effects are treated largely as matters of potential exclusion from digital services, or as gaps in data. For instance, the Academy of Royal Medical Colleges highlights the possible problem of the wealthiest gaining access to the most advanced technologies; the NHS guidelines express concern that AI could be less effective for some ethnic groups if their data isn’t included in early training datasets.

In terms of the wider sociological issues that we are concerned about, regarding the power of AI technology to shape social arrangements in particular way, as Hagendorff (2020) found when looking at general AI ethics frameworks, there was limited evidence that these issues had been considered in these Healthcare-specific ethical AI guidelines. While the impact of AI technologies on institutions and wider social structures is acknowledged in a number of the ethical guidelines, discussion tended to be limited to considerations of the impact on individuals within the healthcare system and on the changes needed to healthcare institutions to facilitate the adoption of AI technologies, rather than the impact on any wider social or democratic structures. For instance, both the Wellcome Trust and the Academy of Royal Medical Societies reports describe how AI is likely to result in the reorganisation of health care systems, with the Academies describing it as a “game-changer for healthcare”. The Academy of Royal Medical Societies paints two possible futures—a utopian world where health inequalities are reduced through improved access to and quality of care; and a dystopian one, in which health inequalities increase, as the healthcare system becomes overwhelmed by the worried well or because healthcare becomes accessible only to the wealthy. The report also acknowledges that these healthcare systems sit within wider infrastructures, and that which of these futures we take will be affected by certain democratic powers, adding that it is now up to “policymakers, politicians, legislators, clinicians and ethicists to decide now how the wider healthcare system will be AI enabled and improved for future generations”. However, no further discussion or recommendations relating to these democratic powers, nor of the institutional or societal effects outside the healthcare systems of the scale that is being anticipated elsewhere in the literature (and discussed in this paper later) were evident, beyond highlighting the possible need to retrain staff or to develop backup plans to facilitate the introduction of these technologies. Specifically, there was no discussion of the role of technology developers, democratic structures or wider society in determining these technological futures.

Similarly, when the wider economic implications of AI are considered, this is also framed narrowly within the context of healthcare. The financial implications of AI on healthcare are typically seen in positive terms—the potential to reduce costs or to exploit data. For instance, the Academy of Royal Medical Societies' report highlights the potential to generate income by selling NHS data and to bring about cost savings via efficiency and streamlined services. The key downside of AI is seen to be the potential to drive new demand as access becomes easier. The Academies of Royal Medical Societies report flags up the potential for AI to exacerbate health inequalities, but this is seen as a concern for individuals in case a two-tier system emerged in which additional payment were required to access the AI enabled ‘higher standard of service’. The wider economic effects of AI on healthcare services—the way in which they tend to centralise services and the potential to drive health inequalities further (discussed further later) are not taken into consideration anywhere in these reports.

The impact of AI on future generations, and its potential to lock us into a particular trajectory, is addressed largely in the context of environmental sustainability. For example, the impact of AI on future generations is highlighted as an issue of concern in the EIT Health report, which refers to calls for AI systems to benefit all human beings, including future generations, in the principles produced by the European Commission High-Level Group on Societal and Environmental Well-being. The UNESCO report also includes environmental sustainability as one of the key ethical issues for AI in healthcare, detailing how information technologies are big producers of greenhouse gases and other waste products throughout their lifecycles, highlighting how the pollution produced has the potential to impact health and setting the International Energy Agency, environmental protection agencies and the WHO the challenge of coordinating efforts to reduce energy use in Big Data management and to avoid e-waste impacting health. Only the UNESCO report included specific recommendations relating to this, however. In contrast, the EIT report indicated that environmental sustainability had not been raised as an important consideration for the technology companies they had consulted.

Overall then, current guidelines for the use of AI in healthcare tend to focus around four of the five concerns identified in the previous reviews of AI ethical guidelines: transparency; fairness; responsibility; and privacy. Similarly, they take a rights-based approach and consider the impact of AI technologies on the individual, underpinned by an assumption that ethically-sound AI in healthcare is possible. They therefore give normative statements about what ethical AI would look like. AI in healthcare is largely seen as a beneficial development, with possible downsides including: Increased demand for services; the potential of two-tier service and growing health inequalities of AI services are charged for; environmental impact. Group effects are seen as the accumulation of individual effects, and the possibility that technologies can exert effects anew at different scales appears to be absent. Ethical issues are also framed within the impact on healthcare. Wider sociological issues were neglected.

## The Multiscale Ethics Framework

### Development and Testing of the Multiscale Ethics Framework

Having identified the gaps in currently existing guidelines for AI Ethics in Health, we set out to develop a new framework that would account for these sociological and political effects that had been identified through our engagement over the past 20 years with literature from science and technology studies and political science (summarised in the introduction above). The idea of scale emerged as a way to order and analyse the effects of technologies from this deep reading of such literature, drawing on key disciplinary ideas within Science and Technology Studies (STS) in particular, along with our reflections on the GP at Hand case study and a number of interdisciplinary discussions with a systems biologist who was thinking about scale as a way to organise complexity within living organisms.

The questions within the framework again drew upon the STS literature described above, as well as the broader ideas that form the ‘canon’ of questions we consider and teach in that field, as well as the issues raised in this case study. The draft framework was then tested and refined—firstly through the two-monthly meetings of the Alan Turning Institute’s Data Ethics Group, throughout 2018 and 2019; secondly through a series of meetings with senior staff from NHS who were developing guidelines for ethical use of data in the development of technologies for the NHS, and with three meetings with clinicians involved in implementing advanced technologies; finally the refined framework was tested by a third party researcher on COVID-19 vaccine passports (O’Donovan et al., 2021a, 2021b) and care-home deaths (O’Donovan et al., 2021a, 2021b), as part of the UK-Pandemic Ethics Accelerator project (UK Pandemic Ethics Accelerator, 2022).

**Fig. 1:**
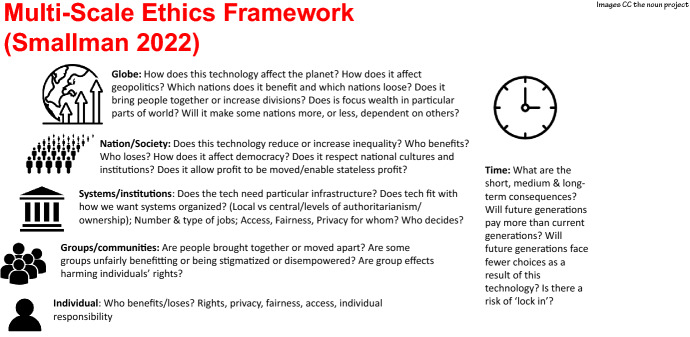
The Multiscale Ethics Framework

Using the idea of scale and drawing on the literature described above, the Multiscale Ethics Framework (Fig. 1  below) acknowledges that risks and benefits are not spread evenly and introduces a topography of impacts into ethical evaluations of technologies, providing a structure that helps technology developers and commissioners to zoom in and out of the issues at stake at different scales, encouraging them to consider the wider sociological effects of their technologies alongside ‘traditional’ ethical concerns affecting the individual.

The framework achieves this by guiding us to consider the impact of their technologies on the scale of (1) the individual (2) different groups and communities (3) the Institution/NHS (4) the Nation (5) Globally (6) Over time, thus forcing the focus beyond the individual.

The questions presented in the figure at each scale reflect a summary of the key issues identified in the literature to date, and aims to provide provocations and discussion points that will encourage reflection, consideration, anticipation and perhaps adjustment, rather than to step towards normative statements of ‘ethical AI’. The scale provided is not necessarily a hierarchy in the sense that things get more important as you move up the scale and does not aim to give a weighting to one scale or another. Instead, it encourages the user to consider whether or not the effects get more significant as the units of assessment become greater, and where the most focus for concern or action is needed.

Added to that, the framework also asks us to consider any interactions between the scales that might be generated by this technology. For instance, individual effects of the technology could create new impact for unexpected groups or communities, such as over-surveillance of certain communities as a result of aggregating data, or the negotiation that might be needed between individual and family privacy.

Finally, acknowledging that the impacts of future technologies are uncertain and often contingent and emerging, and to avoid the risk of this framework becoming part of a compliance culture that sees ethics as something that can be ‘done’, the framework puts forward a series of questions to be considered and reflected upon (and perhaps revisited), instead of the set of normative statements found in more ‘traditional’ ethical frameworks.

Staying with the example of my local health authority, the wider story of the impact of an app and any assessment of whether it is has a positive or negative effect, is tricky because the impact of an AI app like ‘GP At Hand’ varies depending on the position from which you view it: At its simplest, for some time-pressed individuals, the app is a very positive development, enabling them to have a GP consultation without leaving their home or office; to my community, it is associated with a withdrawal of face to face services and concerns about fair access to services; for the NHS, it raises questions affordability and of the appropriateness of a geographical approach to budgeting, if services can now be provided to anyone anywhere; broadening it out to the nation, AI apps opens debates about privatisation of NHS services and how it is funded; and considering time, issues of intergenerational fairness and long-term sustainability need to be taken into account.

Looking at the ethical implications of healthcare technologies at multiple scales would help unearth these wider effects and enable ethical evaluations to account for the possibility that the effects of technologies can look very different from different standpoints; that the risks and benefits of technologies are often uncertain, tend to pattern; and that the ethical impacts include profound effects on social, institutional and democratic arrangements. For example, on an individual level, questions of how these technologies affect patients’ rights, their privacy and fair access to services are pertinent. But event at a community level, questions of the kind of world we are building come into play—does the technology bring people together? Are some groups unfairly benefitting or being stigmatized or disempowered? Moving up the scale, thinking about the impact of the technology at a systems or institutional level raises questions about whether the tech needs particular infrastructure or regime and whether this fits with how we want systems organized. At a National level, we would ask questions about how the benefits and risks are spread, whether the technology affects democracy and the national economy, and does it respect national cultures and institutions? And at the highest scales, we need to ask how the technologies affect the sustainability of the planet, geopolitics and the extent to which they lock us in to particular future arrangements. By mapping impacts at the various scales, the range of possible concerns, risks and the potential tradeoffs ahead become clearer.

## Conclusion

AI and advanced digital technologies are set to transform healthcare and healthcare systems in a significant way in the twenty-first century, with the risks and benefits not patterning equally across individuals, communities and nations. Looking at the existing frameworks for governing ethical AI, I have highlighted how a rights-based approach has focused our gaze on the individual, but the potential of advanced technologies to impact negatively on the very democratic institutions these frameworks are seeking to protect, calls on us to look for new ways of thinking about ethics in the age of AI and data science.

In response to this, I have put forward the Multiscale Ethics (MSE) Framework that aims to bring the wider ethical effects of AI into sharp focus in ethical evaluations. Instead of these wider social and ethical questions being ‘political’ matters that can be sidestepped in the design of ethical AI, if we are to design and implement advanced technologies to work effectively within and enhance the capacity of particular healthcare systems and democratic arrangements, they are pre-requisites to technology design. This approach builds on our earlier work with Responsible Research and Innovation (Groves, 2017; Owen et al., 2012; RRI Tools, 2016; Smallman, 2018), which similarly attempted to bring in social and political implications of emerging technologies into assessments of their accountability, and to use reflexivity and questioning to move away from the tendency of normative statements to be associated with compliance. However, by incorporating these issues into one framework and using scale rather than discipline to structure reflection, the need to create new pathways and processes for technology assessment becomes redundant. The Multiscale Ethics Framework enables these wider effects to be accounted for within the already very well-established ethical evaluation processes.

Further research using the framework with additional case studies will help develop the map of issues being raised by advanced technologies and also refine the framework further. For instance, case studies beyond the UK’s nationalized healthcare system will be interesting in exploring the tensions at play within the scale of ‘institution’—as the interests of public and private organisations come to the fore, as well as between private institutions and individual citizens, in a context when the ethical advantages of collectivity are perhaps less powerful.

However, it remains likely—and frustratingly so perhaps—that many of the questions presented in the Multi-Scale Ethics Framework cannot be answered. Nor do they offer a route to compliance or enabling technological advancement. In fact, in many instances they are likely to bring forward an uncomfortable reflection on what is the purpose of ethical evaluations and guidelines. But maybe that is what is required. If we have the development of a democratic, more equitable and less conflict-ridden future in our sights, it seems vital to ask how we can account for the ways in which advanced technologies affect groups and communities, and structure institutions and societies. Rather than seeing technological advancement as inevitable, or inevitably good, the MSE Framework will enable us to map out the way in which benefits and risks pattern, anticipate some of the more wide-reaching consequences of advanced technologies and make decisions about whether the kind of world we are building with these technologies is the kind of world in which people—like my neighbours—thrive.

## Appendix 1: Documents included in the analysis of current guidelines for ethical AI in Healthcare

- Nuffield Council on Bioethics: The collection, linking and use of data in biomedical research and health care: ethical issues (2015).
- UNESCO International Bioethics Committee Report on Big Data and Health (2017)
- The Wellcome Trust: Ethical, social and political challenges of Artificial Intelligence in Health (Future Advocacy, 2018)
- Digital Europe/Microsoft: Healthcare, AI, Data and Ethics: A 2030 Vision (Digital Europe/Microsoft, 2018)
- Academy of Medical Royal Colleges: Artificial Intelligence in Healthcare (Academy of Royal Medical Colleges, 2019)
- UK Department of Health & Social Care Code of conduct for data-driven health and care technology, (July 2019)
- EIT Health: AI ethics in Health and Innovation (EIT Health, 2019)

## Footnotes

1 I am using the terms AI, data science and advanced technologies to refer to the collective of advanced digital technologies that are currently being developed and promoted within healthcare, rather than any one technology. In the current use, this is referring to ‘narrow’, rather than ‘general’ AI.
